# ST‐elevation myocardial infarction after COVID‐19 infection in a young man with Kawasaki disease

**DOI:** 10.1002/ccr3.6526

**Published:** 2022-11-19

**Authors:** Yuro Matsunaga, Daisuke Fukamachi, Yasuo Okumura, Naoya Matsumoto

**Affiliations:** ^1^ Department of Cardiology Nihon University Hospital Tokyo Japan; ^2^ Division of Cardiology Department of Medicine Nihon University Itabashi Hospital Tokyo Japan

**Keywords:** COVID‐19, Kawasaki disease, ST‐elevation myocardial infarction

## Abstract

A 22‐year‐old male with a history of Kawasaki disease was admitted to our emergency department with an inferior ST elevation myocardial infarction (STEMI). He was infected with COVID‐19 one month prior to admission, and thereafter, stopped the medication 10 days prior to the STEMI.

## Graphical abstract

The case has meticulously highlighted the need for the continuation of antithrombotic therapy in patients with a concomitance of Kawasaki Disease and COVID‐19 infection, which put patients at risk of thrombosis.

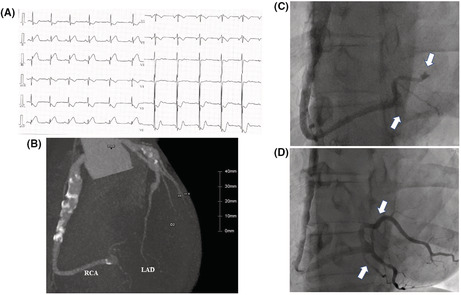

A 22‐year‐old man with a history of Kawasaki disease was admitted to our emergency department with an inferior ST‐elevation myocardial infarction (STEMI) (Figure 1A). An annual checkup coronary CT revealed a coronary aneurysm in both the left and right coronary arteries (Figure 1B) under warfarin and dual antiplatelet therapy. He was infected with COVID‐19 1 month before admission and, after that, stopped the medication 10 days before the STEMI on his own decision. Emergent cardiac catheterization revealed a distal right coronary artery thrombus occlusion (Figure 1C). The coronary flow recovered 2 weeks after plain‐old balloon angioplasty and antithrombotic therapy (Figure 1D). Coronary aneurysms associated with Kawasaki disease are prone to thrombus formation.^1^ In addition, the risk of developing an acute myocardial infarction within one‐month post‐COVID‐19 infection is 5.9 times higher than that without COVID‐19.^2^ This case highlighted the importance of antithrombotic therapy adherence in patients with Kawasaki disease with COVID‐19 infections.

**FIGURE 1:**
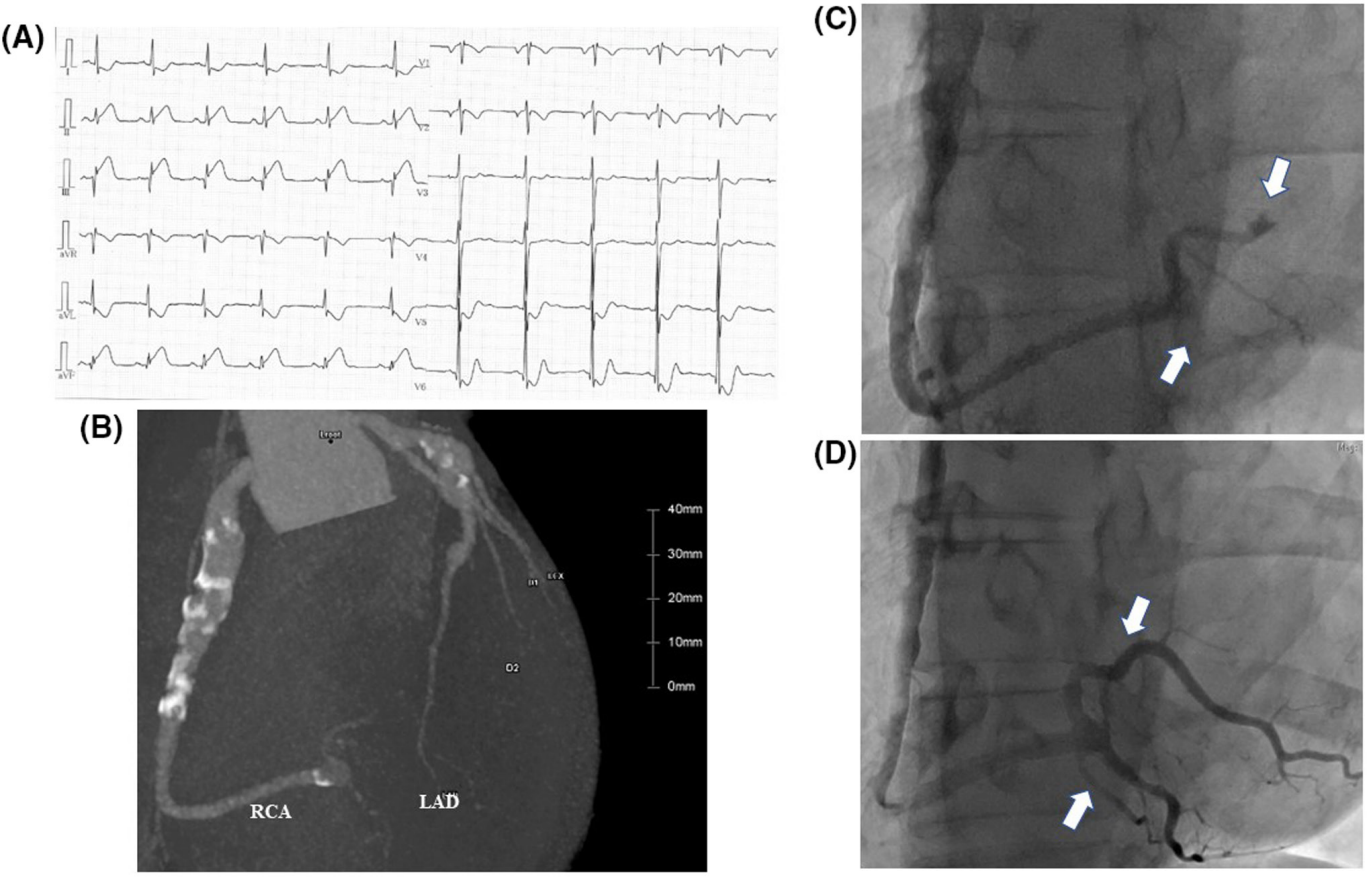
(A) Electrogram shows ST elevation in leads II, III, and aVF. (B) Coronary CT angiography at a regular medical checkup. (C) Coronary angiography revealed a thrombus occlusion in the distal right coronary artery (White arrows). (D) Coronary angiography revealed the disappearance of the thrombus in the distal right coronary artery (White arrows).

## AUTHOR CONTRIBUTIONS

Yuro Matsunaga and Daisuke Fukamachi contributed to the overall management of the case and obtained patient consent. Yasuo Okumura and Naoya Matsumoto contributed to the revision of the paper and advice for treatment.

## CONFLICT OF INTERESTS

We have no potential conflicts of interest related to this manuscript.

## CONSENT

Written informed consent was obtained from the patient to publish this report in accordance with the journal's patient consent policy.

## Data Availability

The data that support the findings of this study are available on request from the corresponding author. The data are not publicly available due to privacy or ethical restrictions.
